# Cryptosporidiosis and Filtration of Water from Loch Lomond, Scotland

**DOI:** 10.3201/eid1401.070562

**Published:** 2008-01

**Authors:** Kevin G.J. Pollock, David Young, Huw V. Smith, Colin N. Ramsay

**Affiliations:** * Health Protection Scotland, Glasgow, Scotland, UK; † University of Strathclyde, Glasgow, Scotland, UK; ‡ Scottish Parasite Diagnostic Laboratory, Glasgow, Scotland, UK

**Keywords:** fresh water, *Cryptosporidium*, filtration, cryptosporidiosis, Scotland, research

## Abstract

Previous evidence has suggested an association between consumption of unfiltered water from Loch Lomond, Scotland, and cryptosporidiosis. Before November 1999, this water had been only microstrained and disinfected with chlorine; however, since that time, physical treatment of the water (coagulation, rapid gravity filtration) has been added. To determine risk factors, including drinking water, for cryptosporidiosis, we analyzed data on laboratory-confirmed cases of cryptosporidiosis collected from 1997 through 2003. We identified an association between the incidence of cryptosporidiosis and unfiltered drinking water supplied to the home. The association supports the view that adding a filtration system to minimally treated water can substantially reduce the number of confirmed cryptosporidiosis cases.

## Abstract 2

Coagulation and rapid gravity filtration coincided with a significant reduction in cryptosporidiosis cases.

Cryptosporidiosis is caused by >1 species or genotypes of the genus *Cryptosporidium*; the most important human pathogens are *C. hominis* and *C. parvum*. *C. hominis* infection is mainly restricted to humans; *C. parvum* infects a variety of mammals (especially neonatal cattle and sheep) as well as humans. Cryptosporidiosis is associated with bloating, abdominal pain, nausea, and diarrhea, which can be profuse and prolonged. Although the illness is normally self-limiting, a recent study showed that it can lead to serious health sequelae (*1*). Moreover, a more severe form of the disease, which may be prolonged or even fatal, may develop in persons with poor immune systems (*2*).

Infection is frequently disseminated by person-to-person transmission, by animals, and indirectly through the environment (particularly by water). Persons at increased risk for *Cryptosporidium* infection include household and family contacts, sex partners of infected patients, users of communal swimming pools, and travelers to disease-endemic regions. Most instances of person-to-person transmission occur directly by the fecal-oral route or indirectly by fomites. Zoonotic transmission from cattle and sheep to humans is a recognized mode of infection for this pathogen; these animals are an important reservoir of human disease (*3*).

Drinking water contaminated with *Cryptosporidium* oocysts is an internationally recognized risk factor for human illness (*4*–*6*); contamination can arise from a variety of sources (*7*) including oocysts from infected humans, livestock, and feral animals present in the catchment. Oocysts remain infectious in the environment and water for prolonged periods and are resistant to most disinfectants used to treat drinking water. Thus, inadequate treatment of drinking water can permit infectious oocysts to be transmitted to susceptible consumers of that water (*8*–*10*).

The need to control microbiologic contaminants in potable waters is dictated by the quality of the source water and requires an integrated multiple-barrier approach. The ability of treatment processes to remove oocysts ultimately depends on the abundance of oocysts in the raw water source and the nature of the treatment processes. For example, the introduction of rapid gravity filtration has been shown to result in ≈3 log_10_ removal of *Cryptosporidium* oocysts (*1*).

In Scotland, 600–900 laboratory-confirmed cases of cryptosporidiosis are reported to Health Protection Scotland (HPS) each year. Within the past 7 years, 2 large outbreaks of water-borne cryptosporidiosis have occurred in Scotland: 90 confirmed cases in Glasgow in 2000 associated with unfiltered Loch Katrine water (*11*) and 140 cases in Aberdeen in 2002 associated with suboptimal filtration of River Dee water. Outbreaks have also occurred in central Scotland; circumstantial evidence suggested an association with minimally treated water from Loch Lomond, particularly in 1998 (*12*,*13*).

Loch Lomond supplies water to ≈34% of the population of central Scotland. Before November 1999, this water was only microstrained (only particles >23 μm were filtered out) and disinfected with chlorine, and the risk of transmitting *Cryptosporidium* spp*.* oocysts (4–6 μm) to consumers of this water was relatively high. In November 1999, enhanced physical treatment (coagulation and rapid gravity filtration), designed in part to reduce the number of oocysts, was introduced. During this time, no substantial changes were made to water treatment for other sources supplying central Scotland. This treatment of the Loch Lomond supply was expected to reduce the oocyst load in the final supply.

To determine the validity of a preliminary study about cryptosporidiosis and water from Loch Lomond (*14*) and to maximize the robustness of the results, we collected data from the entire area supplied with drinking water from Loch Lomond. Our hypothesis was that if a proportion of cases of illness were attributable to drinking unfiltered Loch Lomond water, then illness incidence should be reduced after the introduction of enhanced water treatment. The null hypothesis was that no association would be noted between the enhanced treatment of the Loch Lomond supply and incidence of cryptosporidiosis.

## Methods

The design was a retrospective cohort study of microbiologically confirmed cases of cryptosporidiosis among residents of central Scotland. The period of study (1997–2003) was chosen to enable the detection of any epidemiologic trends in cases and in exposure to risk factors. To calculate the odds of becoming ill if the home water was supplied by Loch Lomond water or another source, we analyzed information on all laboratory-confirmed cases in each of the 5 areas of the National Health Service for Scotland (NHS) Board (Argyll & Clyde, Greater Glasgow, Lanarkshire, Lothian, and Forth Valley). Cases from Forth Valley were excluded, however, because of incomplete data. To improve the potential power of the analysis and to improve our ability to detect a true difference, if one existed, we pooled all cases.

Original NHS Board case investigation records were reviewed, and the following were entered into an Excel database: age, sex, home address postal code, residence, date and age at illness onset, age at time of diagnosis, and history of risk factors (travel [foreign and domestic], recreational water contact, pet ownership, farm animal and farmland contact, and tap water consumption). Using postal codes, we linked cases to the drinking water supply zone and the water source, details of which were supplied by Scottish Water. For analysis purposes, case-patients were divided into 2 groups according to the source of drinking water at the home address (Loch Lomond or other sources). We used NHS Board resident population numbers for 2002, obtained from the General Register Office for Scotland, to identify the population at risk and calculate incidence rates.

Descriptive statistics were used to describe the characteristics of case-patients and the distribution of cases by water source, before and after the introduction of enhanced physical treatment at Loch Lomond. Cases associated with different water sources were compared by using Mann-Whitney tests for quantitative variables and χ^2^ tests for qualitative variables. A χ^2^ test was then performed to investigate any difference in the distribution of cases in persons who received water from Loch Lomond or from other sources before and after the introduction of enhanced treatment of Loch Lomond water. The association with other possible risk factors was investigated by using logistic regression analysis to quantify the characteristics of Loch Lomond case-patients. Unless otherwise stated, we used Minitab statistical software, version 14 (www.minitab.com) at a significance level of 5% for all analyses.

## Results

Of the 2,501 reported cases, 10 had no date of diagnosis and were not further analyzed, which left 2,491 cases in the study. The period incidence of cases in patients who received water from Loch Lomond was 12.8 cases per 100,000 population before filtration (November 1999) and 6.5 per 100,000 after filtration. The period incidence of cases in patients who received water from other sources was 27.7 per 100,000 before November 1999 and 46.9 per 100,000 after November 1999. The period incidence for cases in the rest of Scotland was 39.1 per 100,000 before November 1999 and 66.1 per 100,000 after November 1999. Hence, when the incidence in Scotland as a whole increased considerably, the incidence of cases in the Loch Lomond supply zone decreased by half.

Before filtration, most case-patients were 5–14 years of age, whether they received water from Loch Lomond (62.5%) or from other sources (48.3%) (Table 1). After filtration was introduced to the Loch Lomond supply, the percentage of case-patients 5–14 years of age who received water from this supply decreased (35.1%); however, the percentage in this age group who received water from other sources (29%) also decreased. The population of case-patients 0–4 years of age increased from 5% for the Loch Lomond supply area to 31.6% and from 4.4% to 23.4% for the other-sources area. Age data for 27 case-patients were incomplete.

**Table 1:** Age of cryptosporidiosis case-patients, Loch Lomond, Scotland

Age, y	Loch Lomond, no. (%)			Other, no. (%)		All (n = 2,464)
	Before (n = 341)	After (n = 171)		Before (n = 726)	After (n = 1,226)	
<4	17 (5.0)	54 (31.6)		32 (4.4)	287 (23.4)	390
5–14	213 (62.5)	60 (35.1)		351 (48.3)	356 (29.0)	980
15–44	93 (27.3)	43 (25.2)		283 (39.0)	464 (37.8)	883
45–64	9 (2.6)	10 (5.8)		36 (5.0)	84 (6.9)	139
>65	9 (2.6)	4 (2.3)		24 (3.3)	35 (2.9)	72

Age distributions of case-patients who received water from each of the 2 sources differed significantly (p<0.001). The mean age of case-patients in the Loch Lomond supply area was 16.1 years before filtration was introduced and 16.4 thereafter; for the other supply area, the mean age of case-patients was 20.5 years before filtration and 20.2 thereafter (Table 2).

**Table 2:** Age distribution of cryptosporidiosis case-patients, Loch Lomond, Scotland

Water source	Relation to enhanced water filtration*	No.	Mean, y	Median, y	Range, y
Loch Lomond	Before	341	16.14	8.0	3–92
	After	171	16.43	8.0	1–72
Other	Before	726	20.50	13.0	3–99
	After	1,226	20.24	13.0	0–88

*Coagulation and rapid gravity filtration, introduced in November 1999.

Sex distribution also differed significantly (χ^2^ p<0.001). Of those case-patients living in the Loch Lomond supply zone, most (57.8%) were male; of those outside of the Loch Lomond supply zone, only 48.8% were male. With regard to sex of the study population before and after enhanced water treatment, the sex difference persisted but was reduced after water treatment (Table 3).

**Table 3:** Sex distribution of cryptosporidiosis case-patients, Loch Lomond, Scotland

Water source	Relation to enhanced water filtration*	Male, no. (%)	Female, no. (%)
Loch Lomond	Before	197 (57.8)	144 (42.2)
	After	92 (53.8)	79 (46.2)
Other	Before	354 (48.8)	372 (51.2)
	After	581 (47.4)	646 (52.6)
Total		1,224	1,241

*Coagulation and rapid gravity filtration, introduced in November 1999.

Before enhanced treatment of the Loch Lomond water supply, 1,068 cases were microbiologically confirmed; after enhanced treatment, 1,398 cases were confirmed. Within this cohort, 512 (21%) case-patients lived in the area served by Loch Lomond and 1,954 (79%) lived in areas served by other water sources. For areas not supplied by Loch Lomond water, 37% of cases occurred before enhanced water treatment compared with 63% after (Table 4). Data on water source were incomplete for 25 case-patients.

**Table 4:** Distribution of cases by drinking water source before and after the introduction of filtration at Loch Lomond, Scotland

Relation to enhanced water filtration†	Water source, no. (%)*		
	Loch Lomond	Other	All
Before	341 (222)	727 (846)	1,068
After	171 (290)	1,227 (1,108)	1,398
Both	512	1,954	2,466

*Numbers in parentheses are cases expected under the null hypothesis (i.e., no association between water supply and filtration). †Coagulation and rapid gravity filtration, introduced in November 1999.

Comparison of the observed and expected values illustrates that fewer cases occurred in the area supplied by Loch Lomond after enhanced treatment than would have been expected if the change in treatment had no association with the incidence of cases. The analysis shows a significant association between being a case-patient and living in Loch Lomond before filtration was introduced (p<0.001). Therefore, statistical evidence suggests that the incidence of confirmed cases of cryptosporidiosis in the Loch Lomond area was significantly reduced after enhanced treatment was introduced. This reduction was not seen in the areas not served by Loch Lomond or anywhere else in Scotland.

Binary logistic regression was used to assess which reported risk factors were associated with being a case-patient living in the Loch Lomond supply zone (Table 5). Risk factors investigated were exposure to unfiltered Loch Lomond tap water, contact with a case-patient, contact with farm animals, travel outside the water-supply area, ownership of pets, recreational water contact, consumption of tap water, and others. These risk factors were derived from data collected from the standard cryptosporidiosis investigation forms, which are based on current understanding of risk factors for clinical illness. The frequency of exposure to the reported risk factors was consistent for some factors; e.g., contact with another case-patient was consistently cited least often, and tap water consumption was consistently cited most often. However, the variation in reported exposures over the study period generally showed consistent trends; e.g., recreational water contact increased markedly in both groups, as to a lesser extent did camping and contact with pets. Travel outside the area was cited by a very small proportion of Loch Lomond case-patients before November 1999 but increased almost 10-fold thereafter, compared with a 2-fold increase for case-patients who received water from other sources. The results of the univariate analyses are shown in Table 6.

**Table 5:** Exposure to cryptosporidiosis risk factors before and after the introduction of enhanced water filtration at Loch Lomond, Scotland*

Risk factor reported	Loch Lomond, no. (%)			Other, no. (%)	
	Before filtration	After filtration		Before filtration	After filtration
Tap water consumption	296 (95.2)	146 (96.7)		546 (89.5)	962 (95.9)
Recreational water contact	73 (23.5)	89 (59.2)		183 (29.7)	480 (47.2)
Camping	37 (11.9)	33 (22.1)		81 (13.1)	183 (18.0)
Pets	21 (6.8)	12 (8.0)		41 (6.6)	120 (11.9)
Travel outside area	12 (3.9)	55 (36.2)		91 (14.6)	295 (28.8)
Contact with case	7 (2.3)	4 (2.7)		15 (2.4)	19 (1.9)
Contact with farm animals	33 (10.6)	11 (7.3)		105 (17.0)	142 (14.0)

*Enhanced water filtration is coagulation and rapid gravity filtration, introduced in November 1999.

**Table 6:** Reported exposure variables for cryptosporidiosis, central Scotland

Variable	p value
Exposure to unfiltered Loch Lomond water	<0.001
Consumption of tap water	0.082
Contact with a case-patient	0.694
Camping	0.587
Water contact	0.028
Pets	0.074
Contact with farm animals	0.002
Travel outside water-supply area	<0.001

The factors with χ^2^ p values <0.10 were included in a multiple logistic regression model, corrected for age and sex. When we used backward selection by removing the least significant factor at each stage, the factors associated with being a Loch Lomond case-patient were exposure to unfiltered Loch Lomond water (p<0.001), tap water consumption (p = 0.011), and no contact with farm animals (p<0.001). Case-patients who received water from Loch Lomond were more likely to have become ill before filtration of tap water was introduced (odds ratio [OR] 3.5, 95% confidence interval [CI] 2.8–4.4), more likely to have consumed tap water (OR 1.93, 95% CI 1.16–3.18), and less likely to have had contact with farm animals (OR 0.48, 95% CI 0.34–0.68).

Interaction terms were fitted to the model to determine whether the effect of filtration was confounded with either consumption of tap water or farm animal contact. No significant interaction between filtration and either consumption of tap water (p = 0.400) or contact with farm animals (p = 0.554) was noted.

## Discussion

Effective filtration of drinking water substantially reduces the risk that infectious oocysts will pass into treated water, although residual low-level, intermittent contamination is possible. From 1991 through 1993, oocysts of the dimensions of *C. parvum* were detected in the Loch Lomond supply on 20 separate instances, 55% of which occurred in the months of November and December (*13*). The numbers of oocysts detected were low. Another study found that 11% of final treated water contained *Cryptosporidium* spp. oocysts (2–67 oocysts per 1,000 L) (*15*).

To try to eliminate this water-borne pathogen from drinking water supplies, the water industry and public health responses have focused on establishing effective multiple-barrier water treatment systems (*12*). Paradoxically, however, if drinking water transmission is an important route for maintaining immunity, efforts to eliminate *Cryptosporidium* spp*.* from drinking water can result in increased human susceptibility to infection with *Cryptosporidium* spp (*16*,*17*).

Our results imply an ecologic association between illness and living in the area that received water from Loch Lomond. This association supports the view that risk for illness is increased among those living in an area supplied by minimally treated water and concurs with findings from other studies (*5*,*6*,*18*). We also found that for residents of the Loch Lomond supply area, consumption of unfiltered tap water was a significant risk factor. Of additional interest was the reduced likelihood of zoonotic transmission of *Cryptosporidium* spp. to residents of the Loch Lomond supply area because they were less likely to come into contact with farm animals. This finding provides evidence that residents were being exposed to infectious oocysts by drinking minimally treated tap water. Thus, upgrading water treatment, particularly introducing coagulation and rapid gravity filtration systems, can substantially reduce numbers of cryptosporidiosis cases and lends credence to the implementation of similar treatments for other minimally treated drinking water supplies in Scotland (*12*).

Our conclusions are valid only at the population level because the study was based on the home location of case-patients and the water supply to that location. Data on the water consumption patterns of individuals were insufficient for us to be able to comment on exposure. The ecologic study method is widely accepted in environmental epidemiology because opportunities to collect precise environmental exposure data on individuals are limited. Our use of information routinely collected by Scottish Health Boards was a practical means of improving the evidence base for an association between cryptosporidiosis and drinking tap water. Our study adds further weight to this evidence base.

Before November 1999, the incidence of illness in persons who received Loch Lomond water at home was already substantially lower (less than half) than for their counterparts who received water from other sources. Reasons for this difference are unknown. One possible, but unproven, explanation might be that continuous low-level exposure to oocysts in unfiltered water might result in a higher background level of immunity to *Cryptosporidium* spp. among consumers of Loch Lomond water (*16*) and that an unintended consequence of introducing filtration to Loch Lomond might therefore be reduced levels of immunity to *Cryptosporidium* spp. Continued surveillance of *Cryptosporidium* spp. will therefore be necessary to fully understand the long-term consequences of abrogating environmental risk factors.

Another potential confounding factor for this study might have been the 2001 outbreak of foot-and-mouth disease (FMD) (*19*). During this outbreak, reports of human cases of cryptosporidiosis were significantly reduced in southern Scotland where FMD was present. In the rest of Scotland over the same period, the reduction in cases was not significant (*20*). The reduced movement of farm animals and lower numbers of young animals in the countryside during the spring are likely to have reduced the environmental load of oocysts. Restricted public access to the countryside during the outbreak is likely to have reduced the risk for human environmental exposure to oocysts. In the study cohort, some of the reduction in the incidence of illness noted after November 1999 among Loch Lomond zone residents might have been attributable to reduced environmental exposure, associated with the FMD outbreak. However, the risk factor analysis for the Loch Lomond–zone residents indicated that exposure to farm animals was less; hence, any reduced incidence due to this confounder should be less significant than for the residents outside the Loch Lomond zone. The incidence across Scotland over the whole study period after November 1999 was actually greater than before; any effect of the FMD outbreak was temporary. In contrast, the reduction in case incidence in the Loch Lomond zone was sustained after November 1999.

A possible confounding factor may be that some case-patients from Glasgow who did not receive Loch Lomond water received water from another unfiltered supply (Loch Katrine). The variation in incidence among those who received another water supply may have been caused partially by variation in water from Loch Katrine. An outbreak of cryptosporidiosis occurred in 2000 among persons who received water from Loch Katrine (*11*). However, all known outbreak cases were excluded from this study.

In summary, our data strongly suggest that drinking unfiltered tap water from Loch Lomond transmitted *Cryptosporidium* spp*.* at the population level. Upgrading water treatment, and particularly introducing well-operated coagulation and rapid gravity filtration systems, can substantially reduce the numbers of cryptosporidiosis cases. These findings support implementation of similar treatments for other minimally treated drinking water supplies.
